# The first case report of Kyphoscoliotic Ehlers-Danlos syndrome of chinese origin with a novel *PLOD1* gene mutation

**DOI:** 10.1186/s12881-020-01154-3

**Published:** 2020-10-31

**Authors:** Xiaolin Ni, Chenxi Jin, Yan Jiang, Ou Wang, Mei Li, Xiaoping Xing, Weibo Xia

**Affiliations:** Department of Endocrinology, Key Laboratory of Endocrinology National Commission of Health Peking Union College Hospital, Chinese Academy of Medical Sciences, Shuaifuyuan No. 1, Wangfujing street Dongcheng District, Beijing, 100730 China

**Keywords:** Kyphoscoliotic ehlers-danlos syndrome; *PLOD1* gene; mutation; case report

## Abstract

**Background:**

Kyphoscoliotic Ehlers-Danlos syndrome (kEDS) is a rare autosomal recessive connective tissue disorder characterized by progressive kyphoscoliosis, congenital muscular hypotonia, marked joint hypermobility, and severe skin hyperextensibility and fragility. Deficiency of lysyl hydroxylase 1 (LH1) due to mutations of *PLOD1* (procollagen-lysine, 2-oxoglutarate 5-dioxygenase 1) gene has been identified as the pathogenic cause of kEDS (kEDS-PLOD1). Up to now, kEDS-PLOD1 has not been reported among Chinese population.

**Case presentation:**

A 17-year-old Chinese male patient presenting with hypotonia, joint hypermobility and scoliosis was referred to our hospital. After birth, he was found to have severe hypotonia leading to delayed motor development. Subsequently, joint hypermobility, kyphoscoliosis and amblyopia were found. Inguinal hernia was found at age 5 years and closed by surgery. At the same time, he presented with hyperextensible and bruisable velvety skin with widened atrophic scarring after minor trauma. Dislocation of elbow joint was noted at age of 6 years. Orthopedic surgery for correction of kyphoscoliosis was performed at age 10 years. His family history was unremarkable. Physical examination revealed elevated blood pressure. Slight facial dysmorphologies including high palate, epicanthal folds, and down-slanting palpebral fissures were found. He also had blue sclerae with normal hearing. X-rays revealed severe degree of scoliosis and osteopenia. The Echocardiography findings were normal. Laboratory examination revealed a slightly elevated bone turnover. Based on the clinical manifestations presented by our patient, kEDS was suspected. Genetic analysis revealed a novel homozygous missense mutation of *PLOD1* (c.1697 G > A, p.C566Y), confirming the diagnosis of kEDS-PLOD1. The patient was treated with alfacalcidol and nifedipine. Improved physical strength and normal blood pressure were reported after 12-month follow-up.

**Conclusions:**

This is the first case of kEDS-PLOD1 of Chinese origin. We identified one novel mutation of *PLOD1*, extending the mutation spectrum of *PLOD1*. Diagnosis of kEDS-PLOD1 should be considered in patients with congenital hypotonia, progressive kyphoscoliosis, joint hypermobility, and skin hyperextensibility and confirmed by mutation analysis of *PLOD1*.

## Background

Kyphoscoliotic Ehlers-Danlos syndrome (kEDS) (OMIM 225,400, previously EDS type VIA) is a rare autosomal recessive connective tissue disorder characterized by progressive kyphoscoliosis, congenital muscular hypotonia, severe skin hyperextensibility, and marked joint hypermobility and dislocation [1]. In addition, fragility of the skin with abnormal scarring, Marfanoid habitus, hernia, microcornea, facial dysmorphology, blue sclerae, refractive errors, osteopenia, and arterial rupture could also be presented in patients with kEDS [2]. Estimated prevalence is approximately 1 in 100,000 live births [3].

Deficiency of procollagen-lysine, 2-oxoglutarate 5-dioxygenase 1 (PLOD1) or lysyl hydroxylase 1 (LH1) due to mutations of *PLOD1* was identified as pathogenic cause of kEDS [4]. As an enzyme, LH1 plays an important role in hydroxylation of lysyl residuel in Xaa-Lys-Gly. The hydroxylysine residues serve as sites of attachment for carbohydrate units which are essential for the formation of intra- and intermolecular collagen crosslinks [5]. As a result, LH1 deficiency results in underhydroxylation of lysyl residues and underglycosylation of hydroxylysyl residues, leading to impaired collagen crosslinks formation with consequent mechanical instability of the affected tissues [6]. Abnormal urinary excretion pattern of lysyl-pyridinolines (LP) and hydroxylysyl-pyridinolines (HP) cross-links (LP/HP) is a highly sensitive and specific test for LH1 deficiency [6]. In 2012, mutations of a second gene, FK506 binding protein 14 (FKBP14), was identified as another cause of kEDS (kEDS-*FKBP14*) [7]. Although patients with kEDS-*FKBP14* present a clinically overlapping phenotype with PLOD1-related kEDS (kEDS-*PLOD1*), they can still be distinguished by normal LP/HP, presence of myopathy, and hearing impairment [7].

Although more than 40 different mutations have been identified in the *PLOD1* leading to kEDS, only seven different missense disease associated variants have been described (according to the LOVD Gene Homepage for PLOD1 at https://eds.gene.le.ac.uk/home.php?select_db=PLOD1). In addition, mutation of *PLOD1* has not been reported among Chinese population.

In this report, we present the clinical, biochemical, and molecular data of the first Chinese patient diagnosed with kEDS-*PLOD1* due to a novel *PLOD1* missense mutation.

## Case presentation

**Fig. 1:**
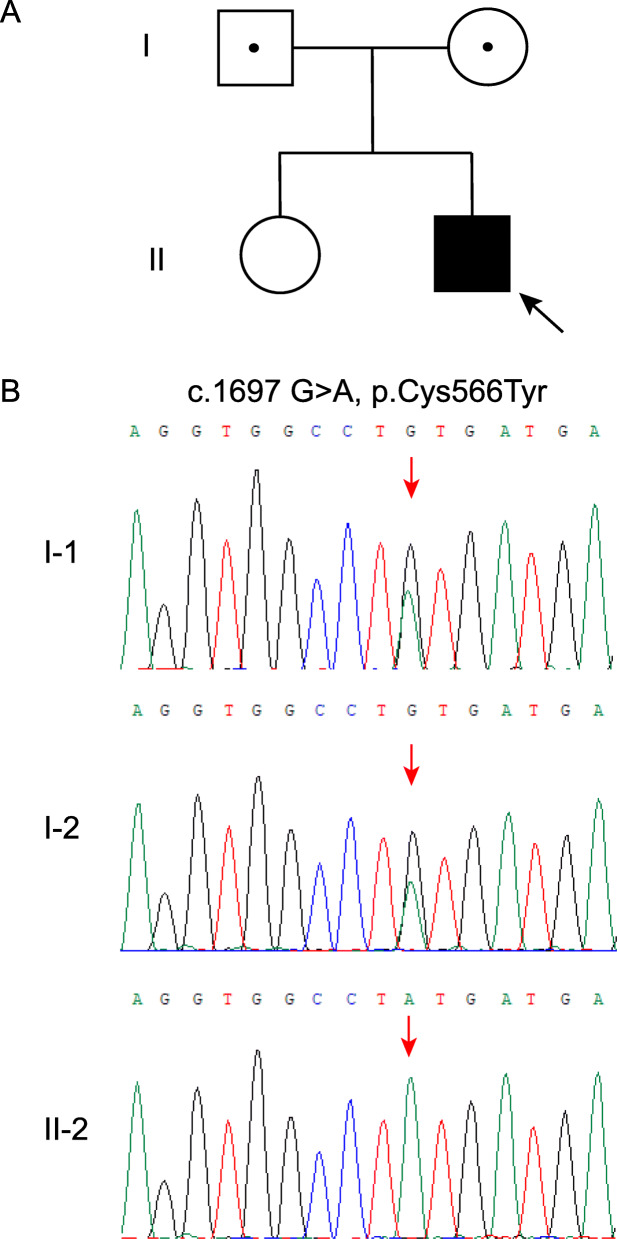
Pedigree and molecular analysis of the family with kEDS-PLOD1. **a** Pedigree of the family of kEDS of Chinese origin. **b** A homozygous mutation of *PLOD1* (c.1697 G > A) in patient (II-2) is a novel missense mutation (p.C566Y). His parents (I-1 and I-2) were heterozygous carriers of corresponding mutation. Black symbols present affected individuals, white symbols with black dots present carriers of the mutation

On October 15, 2019, a 17-year-old patient was referred to our department because of hypotonia, joint hypermobility and scoliosis from childhood. He was the second child of non-consanguineous parents (Fig. 1a), born at term with normal weight (3500 g) and height (55 cm) by normal vaginal delivery. After birth, he was found to have severe hypotonia and did not open his eyes until he was 20 days old. The severe hypotonia led to delayed motor development. He was not able to walk independently until 2 years of age and joint hypermobility was found at that time. Kyphoscoliosis and amblyopia were found at age 3 years. Inguinal hernia was found at age 5 years and closed by surgery. At the same time, he presented with hyperextensible and bruisable velvety skin with widened atrophic scarring after minor trauma. Dislocation of elbow joint has been noted to happen frequently since age of 6 years. Orthopedic surgery for correction of kyphoscoliosis was performed at age 10 years. Kyphoscoliosis reappeared two years after surgery and progressed gradually, but remained stable in the past three years. His intelligence was normal. The patient denied any fracture history. His family history was unremarkable.

**Fig. 2:**
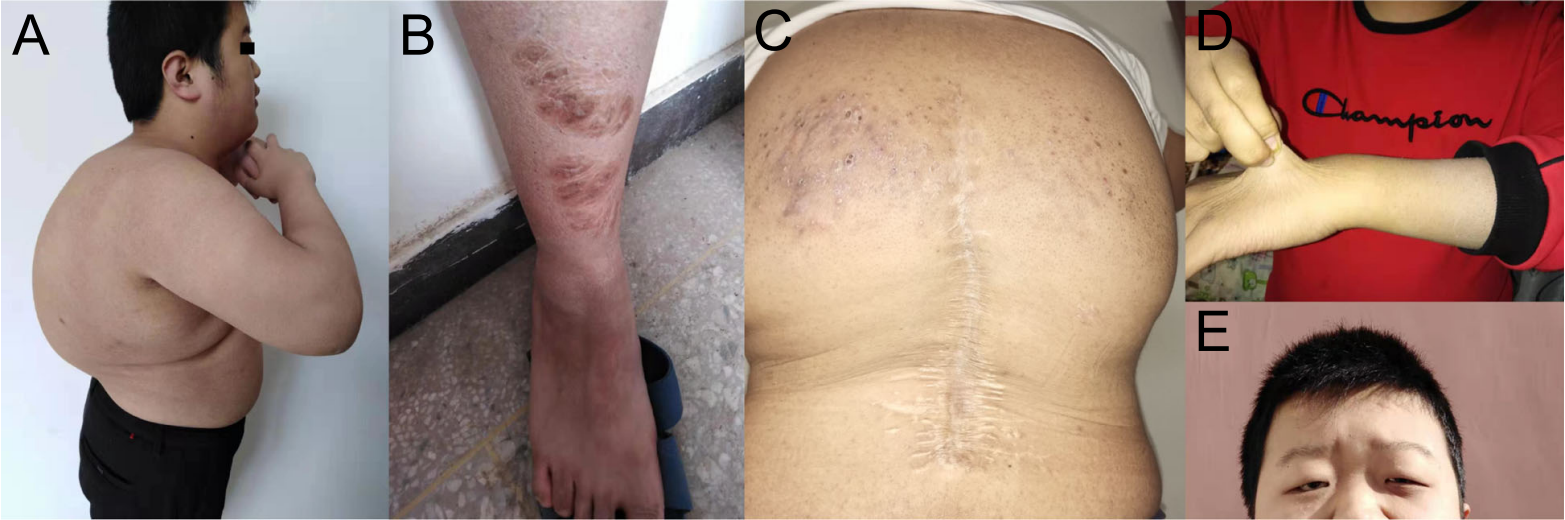
Clinical features of patient at the age of 17 years old. **a** Kyphoscoliosis. **b** Atrophic scars on leg. **c** Scar after surgery on the back. **d** Skin hyperextensibility. **e** Epicanthal folds and down-slanting palpebral fissures

On physical examination at age 17 years, his height and weight were 165 cm (-0.5 SD) and 82.5 kg (+ 1.9 SD), respectively. Blood pressure was 138/94 mmHg without renal artery vascular murmur. He presented with generalized hypotonia, protruding abdomen and kyphoscoliosis (Fig. 2a). He had joint hypermobility (Beighton score 6/9), multiple atrophic scars on the legs (Fig. 2b) and back due to the surgery for correction of kyphoscoliosis (Fig. 2c), and skin hyperextensibility (Fig. 2d). Slight facial dysmorphologies were found, including high palate, epicanthal folds, and down-slanting palpebral fissures (Fig. 2e). He also had blue sclerae. Hearing was normal.

**Fig. 3:**
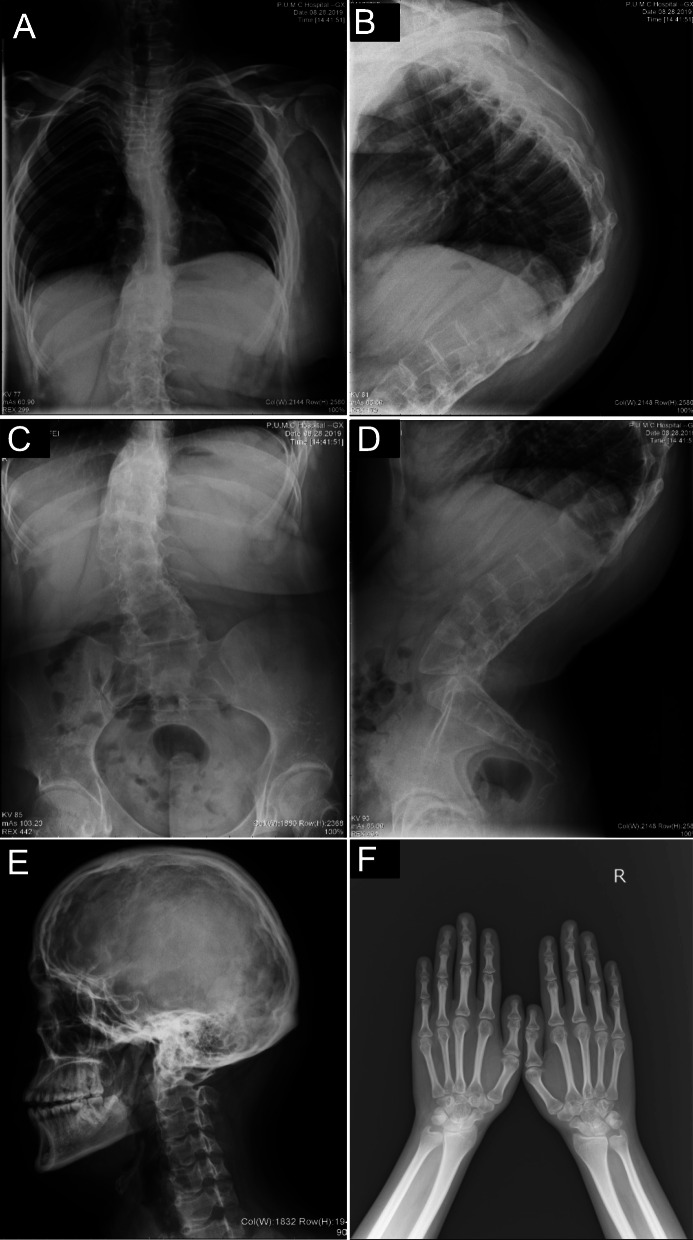
Radiographic findings of the patient at 17 years old. **a** X-ray of spine showing scoliosis and **b** kyphosis of thoracic vertebra, **c** scoliosis and **d** kyphosis of lumbar vertebra. **e** X-ray of skull showing copper-beaten appearance. **f** X-ray of hands showing osteopenia

In order to evaluate bone morphology, X-ray photographs were taken at the age of 17 years old when he visited our hospital. Radiographs of the vertebral column revealed severe degree of scoliosis, concave to the left in the thoracic spine and to the right in the lumbar spine with severe kyphosis (Fig. 3a-d). Radiograph of skull and hands showed copper-beaten appearance and osteopenia, respectively (Fig. 3e and f). The Echocardiography findings were normal, while electrocardiogram revealed sinus tachycardia with 121 bmp. Color Doppler ultrasound of renal artery did not show stenosis. Serum levels of alkaline phosphatase (ALP), N-aminoterminal propeptide of type I procollagen (P1NP), β-isomerized C-terminal telopeptide of type 1 collagen (β-CTX), as well as serum levels of 25-hydroxyvitamin D and intact parathyroid hormone (PTH) were measured. He had both a slightly activated bone resorption (β-CTX 0.96 ng/l, normal range: 0.26 ~ 0.512 ng/ mL) and an activated bone formation (P1NP 89.0 ng/ml, normal range: 15.1 ~ 58.6 ng/ml). Results of regular biochemical analyses revealed high level of uric acid (618 μmol/L, normal range: 210 ~ 416 μmol/L), and free fatty acids (1672 μmol/L, normal range: 129 ~ 769 μmol/L).

Based on the clinical manifestations presented by our patient, kEDS was suspected. Hence, blood samples were obtained from the patient and his parents for genetic analysis. Genomic DNA was extracted from 0.2 ml peripheral blood using QIAamp DNA Blood mini kit (Qiagen, Germany) according to the manufacturer’s protocol. Exons included in coding sequence (CDS) and > 50 bp of exon–intron boundaries of the *PLOD1* and *FKBP14* gene were amplified respectively by polymerase chain reaction (PCR). The amplified products were sequenced by ABI 3730XL sequencer (Applied Biosystems, Foster City, CA). A homozygous mutation involving a G to A transition of *PLOD1* (c.1697 G > A) (NM_000302.4) was detected, resulting in a cysteine to tyrosine substitution at amino acid (p.C566Y) (Fig. 1b). Corresponding heterozygous mutation was detected in his parents. Therefore, the diagnosis of kEDS-PLOD1 was confirmed.

Due to the lack of curative treatment, this patient could only be managed by regular evaluation of musculoskeletal, skin, cardiovascular and ophthalmologic system. Alfacalcidol (0.25ug qd) has been used in our patient to try to improve bone mass and muscle strength with good adherence for 12 months. Diet control and weigh loss had been tried to control the blood pressure. Under the circumstances of COVID-19 pandemic, the patient was followed up regularly by telephone since detailed follow-up to hospital was limited. Due to the poor control of weight reported by patient, nifedipine has been used since September 1, 2020. Recently, the patient reported improved physical strength without adverse events and normal blood pressure with treatment of alfacaicidol and nifedipine by telephone.

## Discussion and conclusions

We describe a 17-year-old patient who presented with congenital muscular hypotonia, progressive kyphoscoliosis, severe skin hyperextensibility and fragility, marked joint hypermobility and dislocation, facial dysmorphology, blue sclerae, refractive errors, and osteopenia. According to the latest revised International EDS criteria, minimal criteria suggestive for kEDS includes the first (congenital muscle hypotonia) and second major criteria (congenital or early-onset kyphoscoliosis), plus third major criteria (Generalized joint hypermobility) or three minor criteria (Table 1) [8]. Our patients met three major criteria and six minor criteria. The diagnosis of kEDS was confirmed by detection of a novel mutation of *PLOD1* gene. To our knowledge, this is the first case of kEDS-PLOD1 of Chinese origin.

**Table 1:** Criteria of kEDS from 2017 International EDS Classification and clinical manifestations of the patient

Criteria	Patient
Major criteria	
Congenital muscle hypotonia	** + **
Congenital or early onset kyphoscoliosis (progressive or nonprogressive)	** + **
Generalized joint hypermobility with dislocations/subluxations (shoulders, hips, and knees in particular)	** + **
Minor criteria	
Skin hyperextensibility	** + **
Easy bruisable skin	** + **
Rupture/aneurysm of a medium-sized artery	**-**
Osteopenia/osteoporosis	** + **
Blue sclerae	** + **
Hernia (umbilical or inguinal)	** + **
Pectus deformity	**-**
Marfanoid habitus	**-**
Talipes equinovarus	**-**
Refractive errors (myopia, hypermetropia)	** + **
Gene-specific minor criteria	
*PLOD1*	
Skin fragility (easy bruising, friable skin, poor wound healing, widened atrophic scarring)	** + **
Scleral and ocular fragility/rupture	** + **
Microcornea	**-**
Facial dysmorphology	** + **
*FKBP14*	
Congenital hearing impairment (sensorineural, conductive, or mixed)	**-**
Follicular hyperkeratosis	**-**
Muscle atrophy	**-**
Bladder diverticula	**-**

kEDS: Kyphoscoliotic Ehlers-Danlos syndrome; PLOD1: procollagen-lysine, 2-oxoglutarate 5-dioxygenase 1; FKBP14: FK506 binding protein 14

Minimal criteria suggestive for kEDS: major criterion (1): congenital muscle hypotonia and major criterion (2): congenital or early-onset kyphoscoliosis plus either major criterion (3): GJH and/or three minor criteria (either general or gene-specific criteria)

Kyphoscoliosisis, mostly accompanied with hypotonia and ligamentous laxity, is usually present at birth or develops in infancy. Although congenital or early onset kyphoscoliosis is obligatory for the diagnosis in the criteria for kEDS, not all of reported patients with kEDS presented with kyphoscoliosis at birth or in infancy [8]. Rohrbach et al. reported that 1 of 12 patients with kEDS did not develop kyphoscoliosis until the age of 27 years. The absence of hypotonia at birth could indicate a more favorable prognosis of not developing spine deformity later in life [6]. Indeed, data from *PLOD1*^−/−^ mice suggested a direct correlation between lack of spine deformity and absence of hypotonia at birth [9]. However, recent study reported two patients who presented to be hypotonic at birth while did not develop congenital or early onset kyphoscoliosis [10]. So, kyphoscoliosis is not necessarily existent in patients with kEDS, and the association between occurrence of kyphoscoliosis and hypotonia at birth is still controversial. It should be noted that the main differences of diagnostic criteria between kEDS and classic EDS (cEDS) are the presence of kyphoscoliosis and hypotonia at birth, while hypotonia could also exist in cEDS [11]. In this condition, patients with kEDS could be easily misdiagnosed as cEDS. In patients who satisfy the main criteria of cEDS without mutataion of COL5A1 and COL5A2, the possibility of *PLOD1* mutation should be considered. In our study, the patient presented to be hypotonic at birth, but kyphoscoliosis was not noted until 3 years of age.

Although vascular incidents are more common in vascular EDS (vEDS), eight cases with antenatal or neonatal vascular incidents have also been reported in kEDS [2, 6, 12–17]. In addition, as a major life-threatening complication in this disorder, vascular incidents such as aortic dilation/dissection and rupture of medium-sized arteries often occurs during transition period from youth to adulthood [14, 15]. Yeowell et al. reported a patient with kEDS who presented with aortic root dilatation and died from arterial rupture [14]. Echocardiography in our patient was normal. Orthostatic intolerance was reported to be prevalent in hypermobile EDS (hEDS) and was most frequently expressed as postural orthostatic tachycardia. Although we did not perform autonomic function testing on our patient, consistent with previous study [18], relatively high resting systolic and diastolic blood pressure and heart rate were found, suggesting increased resting cardiac sympathetic activity. Although the underlying mechanism of vascular incidents has not been well established, the abnormal collagen structure is a facilitating factor. Control of blood pressure was suggested to reduce the risk of arterial rupture [3]. Although nifedipine used in our patient normalized blood pressure successfully, evaluation of cardiovascular structure and function by echocardiography should be followed up regularly [3].

**Fig. 4:**
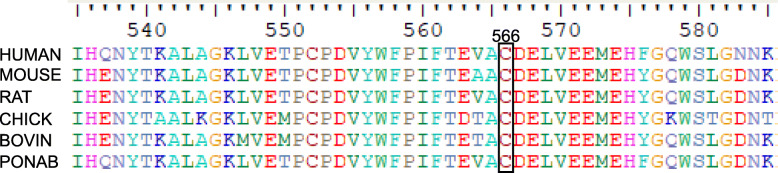
Sequence alignment of human PLOD1 with other species. Number and black box indicates the mutant residue in human PLOD1. The amino acid cysteine at position 566 is highly conserved among different species

At present, 44 different mutations of *PLOD1* (Ehlers Danlos Syndrome Variant Database) has been reported, including missense, nonsense, insertion, duplication, deletion, splicing mutations. Among them, the 8.9 kb duplication of 7 exons (exons 10–16; c.1067_1846 dup) is the most common mutation [19], followed by nonsense mutation (p.Arg319*), while missense mutations are relatively rare. The patient harbored a homozygous missense mutation (c.1697 G > A, p.C566Y) resulting in a cysteine to tyrosine substitution, and his parents were heterozygous carriers of the corresponding mutation. The substituted cysteine was highly conserved in human, rat, mouse, chicken and chimpanzee (Fig. 4). This variant was not reported in several global human genome databases, including the Human Gene Mutation Database (HGMD) (https://www.hgmd.cf.ac.uk/ac/), dbSNP (https://www.ncbi.nlm.nih.gov/SNP), ClinVar database (https://www.ncbi.nlm.nih.gov/clinvar/), 1000 Genomes (https://www.1000genomes.org), the Exome Aggregation Consortium (ExAC) (https://exac.broadinstitute.org). This variant is possibly causative for kEDS because it was predicted to be “PROBABLY DAMAGING” with a score 0.999 by Polymorphism Phenotyping version 2 (PolyPhen-2) (https://genetics.bwh.harvard.edu/pph2/), “AFFECT PROTEIN FUNCTION” with a score 0.00 by Sorting Intolerant From Tolerant (SIFT) (https://sift.bii.a-star.edu.sg), and “disease causing” by Mutation Taster (https://www.mutationtaster.org/). Clinical severity was observed to be variable in individuals with kEDS-PLOD1, even in patients with same mutation [5, 6]. No specific work has been carried out to analyze genotype–phenotype correlations to date [5, 15, 19].

One major limitation of our study is the incomplete follow-up. We will keep evaluating the structure and function of relevant organs and monitoring the development of existed symptoms of our patient regularly.

In conclusion, this is the first case with kEDS-PLOD1 in China. We identified one novel mutation of *PLOD1*, extending the mutation spectrum of *PLOD1*. Diagnosis of kEDS-PLOD1 should be considered in patients with congenital hypotonia, progressive kyphoscoliosis, joint hypermobility, and skin hyperextensibility. The suspected diagnosis could be confirmed by mutation analysis of *PLOD1*. All of these will help avoid unnecessary examination and increase early detection rate.

## Data Availability

The datasets generated during the current study are available in the Mendeley repository, https://dx.doi.org/10.17632/85x3f8r789.1 (https://doi.org/10.17632/85x3f8r789.1).

## Abbreviations

kEDS: Kyphoscoliotic Ehlers-Danlos syndrome
cEDS: Classic EDS
vEDS: Vascular EDS
hEDS: Hypermobile EDS
PLOD1: Procollagen-lysine, 2-oxoglutarate 5-dioxygenase 1
FKBP14: FK506 binding protein 14
LH1: Lysyl hydroxylase 1
LP: Lysyl-pyridinolines
HP: Hydroxylysyl-pyridinolines
ALP: Alkaline phosphatase
P1NP: N-aminoterminal prepeptide of type I procollagen
β-CTX: β-Isomerized C-terminal telopeptide of type 1 collagen
PTH: Parathyroid hormone
